# Medical education across three colleges of medicine: perspectives of medical students

**DOI:** 10.1016/j.heliyon.2022.e11426

**Published:** 2022-11-05

**Authors:** Qasim A. El-Dwairi, Intisar Mustafeh, Moawiah Khatatbeh, Mohammed I. Malki, Ayman G. Mustafa

**Affiliations:** a Department of Anatomy, Faculty of Medicine, Jordan University of Science and Technology, Irbid, Jordan; b Department of Educational Sciences, College of Education, Qatar University, Doha, Qatar; c School of Health and Environmental Studies, Hamdan Bin Mohammed Smart University, Dubai, United Arab Emirates; d Department of Basic Medical Sciences, College of Medicine, QU Health, Qatar University, Doha, Qatar

**Keywords:** Curriculum, Integration, Jordan, Medical education, Student

## Abstract

**Aim:**

This study aimed to explore and evaluate various components of the medical education process (lectures, labs, small-group discussions, clinical rotations, and undergraduate research) in three colleges of medicine in Jordan.

**Methods:**

This cross-sectional questionnaire-based study included 849 undergraduate students from three main medical colleges in Jordan. Statically valid responses were considered for 684 students. The participants were from Jordan University of Science and Technology, Yarmouk University, and the University of Jordan.

**Results:**

The distribution of students according to their admission status was 276 (40%) regular, 266 (38.9%) parallel, and 142 (20.8%) international programs. Personal interest and self-initiation were the major motives for studying medicine in 66.1%. Regarding the frequency of attending classes, University of Jordan students reported the highest rate of regular classes' attendance (93%). The study also reported that lecture notes and textbooks were the main sources of learning for medical students. The study also reported superior academic performance of students in the regular program compared to students in the parallel and international programs. Participants of the study criticized the medical curricula in the three colleges mentioned above because of the lack of active research programs. Most of the students (40%–56%) also complained that the lectures within the modules were not well-integrated, and they felt that the academic environment was moderate (48–59%). In addition, most students in the clinical phase complained of overcrowding in hospital wards during clinical rotation.

**Conclusions:**

Based on students' feedback, multiple aspects of the medical education process require substantial reform to meet the expectations of medical students in Jordan.

## 1 Introduction

Medicine is considered the most sought-after profession [1]. Jordanian universities house five colleges of medicine, where hundreds of medical students enroll every year. Several quantitative measures indicate the good quality of medical education in Jordan, such as the highly competitive admission to medical schools and the academic performance of admitted medical students during their study years [2]. Furthermore, the performance of medical graduates of Jordanian universities in the USMLE is among the best in the region [3]. The medical education process depends mainly on three variables: students, curricula, and teaching staff [4]. We can add to these variables the interaction between them to create a healthy learning environment.

With regard to medical education in Jordan, drastic changes have been implemented over the last two decades. For example, over the last two decades, medical colleges have switched from a classical subject-based curriculum to an integrated system-based modular curriculum. In addition, some elements of the problem-based curriculum, namely small group discussions of clinical problems, were incorporated into the new curriculum [2]. Such curriculum gives a much more central role for the students in the medical education process [5]. Moreover, such curricular change can be challenging for faculty, students [6], and the overall medical education process. It seems logical that such changes in the medical curriculum require highly competitive medical students to cope with them [7]. Contrary to this and due to the financial distress of universities, parallel and international programs were launched to accept students who were not competitive enough to get admitted in the regular programs [2]. This has led to a vast increase in the number of medical students, regardless of the readiness or capacity of medical colleges. Such changes are expected to affect students' learning behavior and their satisfaction with medical education in their institutions [8]. Therefore, this study was designed to report features of students' learning behavior and their feedback on specific elements of the medical education process within the integrated system-based curriculum. Furthermore, students' responses will be analyzed according to three domains: admission type, university, and level of study.

## 2 Methods

### 2.1 Study design and participants

This cross-sectional study was conducted among undergraduate medical students at three public universities in the north and middle of Jordan using simple random sampling and administering a questionnaire. The study included 684 students from the colleges of medicine at Yarmouk University (YU), Jordan University of Science and Technology (JUST), and the University of Jordan (JU). The inclusion criteria for participation were as follows: being an enrolled undergraduate medical student at any of the three universities, being at second-year level or higher, and being 18 years and older. The Institutional Review Board (IRB, approval number: 514/2015) at Jordan University of Science and Technology approved this study.

### 2.2 Study instruments and variables assessment

A new questionnaire was specifically designed to collect data in this study. The newly drafted questionnaire was presented to a reviewer panel composed of three members for refinement and re-wording of questions to ensure that statements were understandable and meaningful to the participants and that the questionnaire consistently measured what it was intended to measure. The reliability of the survey instrument was determined using an internal consistency method. Before conducting the electronic survey, 30 students from the respective colleges were interviewed and asked to answer a questionnaire to ensure that they understood the included questions. After administering the questionnaire used in this study, the responses to all domains were statistically tested for internal consistency. Cronbach’s alpha coefficients for all domains ranged from 0.70 to 0.85.

The final questionnaire comprised three sections: demographic characteristics of students, general information regarding their study, and students' attitudes towards their colleges. The total number of questionnaire items used was 27.

### 2.3 Ethics approval

The Institutional Review Board of Jordan University of Science and Technology approved the use of a questionnaire to collect data for the study.

### 2.4 Data collection

To eliminate researcher bias, the questionnaire was prepared and sent to students' email addresses through the online systems at the three selected universities, as researchers have direct access to the mailing addresses of all students. After obtaining their consent, participants completed a questionnaire that took approximately 10–15 min to complete.

### 2.5 Statistical analysis

All data were analyzed using SPSS (version 20 for Windows). The frequency distribution and descriptive criteria were calculated. Questionnaire responses were compared using the chi-squared test. A p-value < 0.05 was considered to indicate statistical significance in all cases.

## 3 Results

This cross-sectional study was conducted on 849 undergraduates of three major medical colleges in Jordan. Statistically valid responses were considered for 684 students and the remaining nonvalid responses were excluded from the study. The participants were 294 students from JUST, 218 students from Yarmouk University, and 172 students from the University of Jordan. The distribution of students according to their admission status was 276 (40%) regular, 266 (38.9%) parallel, and 142 (20.8%) international programs. Personal interest and self-initiation were the major motives for studying medicine in 452 (66.1%) students. Other motives were also reported, such as family influence in 208 (30.4%) and social prestige in 24 (3.5%) students. Regarding the distribution of participants according to the level of study, second-year students were 270 (39.5%), third-year students were 173 (25.3%) and fourth-year and above were 241 (35%), Table 1. Cross-tabulation analysis was performed to evaluate the students' responses to the study questionnaire items according to their university (Table 2), level of study (Table 3), and admission category (Table 4). Moreover, the study reported a correlation between students' scores in two courses (general anatomy and general histology) and the admission category (Table 4). Only the statistically significant results are presented in the tables.

**Table 1:** Background information and Characteristics of the study sample (n = 684).

	n (%)
University	
JUST	294 (43.0)
YU	218 (31.9)
JU	172 (25.1)
Level/Year of study	
Second year	270 (39.5)
Third year	173 (25.3)
Fourth to sixth year	241 (35.2)
GPA	
60-69	113 (16.5)
70-79	238 (34.8)
80-89	211 (30.8)
90-99	122 (17.8)
Admission Category	
Regular	276 (40.0)
Parallel	266 (38.9)
International	142 (20.8)
Motive to study medicine	
Self-motivation	452 (66.1)
Family influence	208 (30.4)
Social Prestige	24 (3.5)

JUST: Jordan University of Science and Technology.

YU: Yarmouk University.

JU: Jordan University.

**Table 2:** Students' responses according to their university.

Item	Response	University						p-value
		JUST	%	YU	%	JU	%	
Attendance pattern	Regular	238	81.0	137	62.8	160	93.0	0.000
	Irregular	43	14.6	65	29.8	12	7.0	
	Rare	13	4.4	16	7.3	0	0.0	
Learning sources for exams	Lecture notes only	102	34.7	68	31.2	66	38.4	0.001
	Lecture notes & textbooks	159	54.1	142	65.1	101	58.7	
	Private Tutors	20	6.8	7	3.2	4	2.3	
	Old questions	13	4.4	1	0.5	1	0.6	
Practical sessions enhance my understanding of the relevant topic	No	168	57.1	76	34.9	56	32.6	0.001
	Some of them	86	29.3	64	29.4	64	37.2	
	Most of them	40	13.6	78	35.8	52	30.2	
Instructors keep their lectures up to date (in both content and style)	No	82	27.9	15	6.9	31	18.0	0.000
	Some of them	162	55.1	115	52.8	92	53.5	
	Most of them	50	17.0	88	40.4	49	28.5	
The number of students in each group during clinical rounds	Less than 10	33	11.2	65	29.8	24	14.0	0.000
	10–20	221	75.2	137	62.8	125	72.7	
	More than 20	40	13.6	16	7.3	23	13.4	
Admission category	Regular	96	32.7	118	54.1	62	36.0	0.001
	Parallel	133	45.2	77	35.3	56	32.6	
	International	65	22.1	23	10.6	54	31.4

**Table 3:** Students' responses according to their current level of study.

Item	Response	Level of study						p-value
		2^nd^ year	%	3rd year	%	≥4th year	%	
Lectures are well integrated	No	20	7.4	16	9.2	37	15.4	0.001
	Some of them	98	36.3	87	50.3	87	36.1	
	Most of them	152	56.3	70	40.5	117	48.5	
Learning sources for exams	Lecture notes only	75	27.8	82	47.4	79	32.8	0.000
	Lecture notes & textbooks	177	65.6	81	46.8	144	59.8	
	Tutors	16	5.9	9	5.2	6	2.5	
	Old questions	2	0.7	1	0.6	12	5.0	
Instructors present during their lectures additional information from relevant research articles	No	37	13.7	24	13.9	55	22.8	0.037
	Some of them	167	61.9	109	63.0	142	58.9	
	Most of them	66	24.4	40	23.1	44	18.3	
Instructors keep their lectures up to date (in both content and style)	No	29	10.7	34	19.7	65	27.0	0.000
	Some of them	155	57.4	91	52.6	123	51.0	
	Most of them	86	31.9	48	27.7	53	22.0	
The number of students in each group during your clinical rounds	Less than 10	33	11.2	65	29.8	24	14.0	0.000
	10–20	221	75.2	137	62.8	125	72.7	
	More than 20	40	13.6	16	7.3	23	13.4	
Study pattern	Regular	139	51.5	101	58.4	93	38.6	0.000
	Weekend	55	20.4	32	18.5	77	32.0	
	Before exams	76	28.1	40	23.1	71	29.5	
Satisfaction with the academic environment and facilities in your college	Insufficient	86	31.9	50	28.9	95	39.4	0.035
	Moderate	135	50.0	102	59.0	117	48.5	
	High	49	18.1	21	12.1	29	12.0	
Availability of options to participate in active research in your college	None	161	59.6	85	49.1	163	67.6	0.002
	Occasional	64	23.7	51	29.5	37	15.4	
	Available	45	16.7	37	21.4	41	17.0

**Table 4:** Students' responses according to their admission type.

Item	Response	Nature of admission						p-value
		Regular	%	Parallel	%	International	%	
Lectures are well integrated	No	26	9.4	40	15.0	7	4.9	0.001
	Some of them	119	43.1	106	39.8	47	33.1	
	Most of them	131	47.5	120	45.1	88	62.0	
Instructors keep their lectures up to date (in both content and style)	No	48	17.4	61	22.9	19	13.4	0.016
	Some of them	153	55.4	145	54.5	71	50.0	
	Most of them	75	27.2	60	22.6	52	36.6	
Instructors have positive influence on your academic achievement	No	51	18.5	58	21.8	19	13.4	0.026
	Some of them	109	39.5	119	44.7	76	53.5	
	Most of them	116	42.0	89	33.5	47	33.1	
Grade in General Anatomy	50–59	14	5.1	24	9.0	7	4.9	0.000
	60–69	32	11.6	47	17.7	28	19.7	
	70–79	61	22.1	74	27.8	42	29.6	
	80–89	86	31.2	85	32.0	44	31.0	
	90–99	83	30.1	35	13.2	21	14.8	
	Fail	0	0.0	1	0.4	0	0.0	
Grade in Histology	50–59	8	2.9	19	7.1	6	4.2	0.000
	60–69	38	13.8	49	18.4	26	18.3	
	70–79	65	23.6	98	36.8	53	37.3	
	80–89	102	37.0	72	27.1	43	30.3	
	90–99	63	22.8	26	9.8	14	5.2	
	Fail	0	0.0	2	0.8	0	0.0	
Student GPA	60–69	23	8.3	57	21.4	33	23.2	0.000
	70–79	76	27.5	102	38.3	60	42.3	
	80–89	102	37.0	79	29.7	30	21.1	
	90–99	75	27.2	28	10.5	19	13.4	

University of Jordan (UJ) students reported the highest rate of regular class attendance. High rates of regular attendance were noted for (UJ) 93% followed by 81% (JUST) and 62% of (YU). On the other hand, irregular class attendance was 14.6%, 29.8%, and 7% for the three respective colleges. Regarding the students' study style, using both lecture notes and textbooks was the dominant style of learning among students in the ratios of 58.7%, 54.1%, and 65.1% for UJ, JUST, and YU, respectively. The rates of students depending on lecture notes alone as the main source of studying for exams were 38.4%, 34%. & and 31.2% for the three colleges respectively. 3–6% of the students in the three colleges attended off-campus private tutoring sessions and 1–4% of them depended on old exam questions, Table 2.

Moreover, the study reported feedback from medical students on medical curricula and the medical education process at their respective colleges. It seems that the three colleges mentioned lack active research programs. 59.6%, 49.1%, and 67% of second-, third-, and fourth-year students reported inaccessibility to research activities in the three colleges, Table 3.

The academic staff may not practice updating the medical lectures. 57.4%, 52.6% and 51% of the Second, third- and fourth-year students reported that the lectures were not updated. The majority of the second-, third-, and fourth-year students reported proper integration of lectures (40%-%-56%), whereas 36.1%–50.3% of students reported that few instructors integrated their lectures and 7.4%–15.4% reported no-integration of lectures, Table 3. The majority of the students felt that the academic environment was moderately positive in terms of meeting students' expectations (48–59%), while 28.9%–39.4% of the students feel it was insufficient. The lowest proportion of students (12%–29%) believed that the academic environment was suitable. Most students in the clinical phase (fourth-, fifth-, and sixth-year students) complained of overcrowding in their respective wards during clinical rotations (Table 3).

The study also compared students' performance in two courses, namely general anatomy and general histology. Regular students of the three colleges obtained high marks (A) in anatomy and histology. The anatomy high mark ratio was 30.1 %, 13, .2%, and 14.8% for regular, parallel, and international students, respectively. Histology marks showed a similar ratio patterns of 22.8%, 9.8%, and 5.2% for regular, parallel, and international students respectively, Table 4.

## 4 Discussion

The present work provides a comprehensive evaluation of several elements of the medical education process, including lectures, labs, small-group discussions, clinical rotations, and undergraduate research in three major medical colleges in Jordan. The study sample comprises students from three major colleges of medicine in Jordan (Table1). Specifically, the study reported students' learning behavior and perceptions on various components of the medical education process at their respective universities. The selection of new medical students for admission through regular programs in Jordan is based solely on the results of high school grades. Usually, the top 1% of students are selected in the regular programs. Whereas for the parallel and international programs, students with much lower academic performance are usually admitted. The major limiting factor for admission to parallel and international programs is the financial ability to pay the much higher tuition and fees [2]. Unfortunately, this has led to the admission of many students to medical schools who have never been in the ‘A’ student category. The admission of such marginally qualified students has led to increased failure and dropout among medical students, especially in the first two years [9, 10].

In addition, the increased number of medical students has affected most critical aspects of the medical curriculum. For instance, the huge number of medical students admitted every year has led to a dramatic increase in the academic and administrative workloads of faculty members. Such inflation of the workload prompted the teaching staff to alter and modify many of the essential tools of medical education. For example, many faculty members are now unable to properly monitor student attendance and are unable to follow up with students during regular lectures and labs. This has negatively affected students' class attendance, which is a major determinant of academic performance [11]. In addition, owing to the large number of students within each batch, the number of laboratory sessions was reduced to a minimum. Moreover, in many departments, laboratory sessions have been replaced by compact discs and Internet links to be reviewed by students as a self-study activity. This denied students the proper venue to learn medical sciences and adopt professionalism [12].

Another consequence of the large classes of medical students is their complete reliance on multiple-choice questions in the evaluation process. Even though it is a valid tool for evaluation, limiting the evaluation process to this method has deprived students of developing oral and written communication skills [13]. Moreover, using only the MCQ in the assessment greatly undermined the evaluation of students in laboratory sessions. For instance, MCQs may not be suitable for assessing the learning outcomes of laboratory sessions in subjects, such as anatomy, histology, and pathology. Consequently, there has been a shift towards giving more importance and weight in the final grade to the theoretical aspects at the expense of the practical components. This trend has undermined the ability of practical sessions to bridge the gap between basic and clinical sciences, which is detrimental to the medical education process [14].

The feedback of the students in this study continues to suggest that medical students are the major determinants of their success or failure in medical schools. For example, strongly motivated students who chose to study medicine out of personal interest were highly committed (Tables 2 and 3). This was reflected by their regular class attendance and extensive study using both lecture notes and textbooks (Table2). This is in line with previous studies suggesting the same central role for medical students in their success or failure [15]. Interestingly, the study reported that the majority of regular program students chose medicine out of their personal interest, attended classes regularly, pursued a regular extensive study style, and subsequently excelled in academic performance (Table 4). In contrast, most parallel and international program students did not attend classes regularly. They were also less rigorous in their study style.

Moreover, the study observed that a significant number of students in parallel and international programs only used lecture notes as a source of learning (Table 4). They might also rely on private tutors and samples of old exam questions. These factors negatively influenced their academic performance, as shown in (Table 4). Our results are in line with published articles addressing the positive influence of regular attendance of classes and studying from textbooks on the academic progress of medical students [16, 17]. One suggested way to encourage students to use textbooks is by providing them with instructor-generated notes that guide students through the textbook [18].

Universities in Jordan have been following the old scheme of medical education, namely the subject-based yearly system. A new system-based modular integrative system has been adopted by Jordanian medical schools since the year 2000 [2]. This system has brought about important reforms in medical education [19]. However, they still suffer from certain inadequacies. For example, the system is very compact, and both students and staff suffer from material overload [20]. In addition, instructors do not deliberately plan for integration, which is the core point of this system. Students' feedback clearly shows that only 40%–56% of them believe that faculty members integrate their lectures (Table 3). These facts are mainly due to the lack of training and workshops necessary to prepare faculty members for the challenging task of teaching in an integrated curriculum [5].

Another important aspect of medical education is the delivery of information through lectures. Current medical students, the millennial students, are more receptive to using technology, simulation, and an interactive style of instruction [21, 22]. However, the large number of admitted students compromises the ability of the teaching staff to adopt innovative methods of instruction.

Moreover, the content of lectures should be very dynamic and updated frequently according to scientific discoveries. For example, approximately ten years ago, there was very little focus on cloning and stem cell therapy. Currently, these topics are of great importance worldwide [23]. Unfortunately, as shown in Tables 2 and 3, only 22%–31.9% of students believed that instructors updated their lectures.

Even though the value of research experience for medical students is not arguable [24], the medical curriculum in Jordan does not provide enough room for this important component. Medical research courses, as well as laboratory work, are an integral part of the medical curricula worldwide [25]. Therefore, graduates of medical colleges in Jordan have a very serious deficiency, which can potentially create a barrier to postgraduate studies [26]. Students' involvement in conducting and disseminating research should be encouraged. Research has a positive impact on medical training in terms of corroborating evidence-based medicine in medical training programs [27]. Moreover, allowing medical students to conduct research is well-received by them and increases their satisfaction with medical education [28].

The clinical rotation of students in different wards of health units is an integral part of medical education. To ensure proper training, students were divided into groups of suitable numbers. The large size (up to 20 students, as reported in Table 2) of the student groups in clinical rotations is expected to compromise their ability to gain adequate training [29]. Medical trainees should be allotted a specific number of patients in each ward of the hospital to train in taking medical history, investigation, differential diagnosis, and finally a discussion on the diagnosis and proper treatment. This shortcoming is mainly due to the admittance of a large number of students that overcome the available medical unit’s capacities. One suggested solution could be the use of clinical skill laboratories and high-tech simulators to augment clinical training and cover any gaps in clinical rotations [30].

## 5 Conclusions

Studying medicine is a difficult task that requires an appropriate selection process for students and academic staff. While academic background was the main criterion of admission to medical colleges in the past, new admission and selection criteria are needed to evaluate students' personality, ability to withstand stress, and commitment to lifelong medical education.

Students of the regular program are self-initiated, regular in their attendance and studies, use varied recommended references, and achieve higher marks than parallel and international program students. Parallel and international students are less self-initiated, less regular in their attendance and study, and rely more on lecture notes. Therefore, it stands to reason that they obtain fewer marks and have higher dropout rates than their regular classmates.

Moreover, the study findings revealed that students were not satisfied with the delivery of materials. This was obvious by the feedback of a significant proportion of them who believed that faculty members did not deliver integrated lectures, kept the lectures up-to-date, or adopted simulation and interactive instructive styles. It is advisable to provide teaching staff with appropriate training on teaching within integrated modules. Regarding clinical training, the use of clinical skill laboratories may be necessary to mitigate the effect of large groups during clinical rotations. Finally, medical research is an integral component of medical education worldwide, and medical curricula should be reformed to create sufficient room for structured medical research.

## 6 Limitations of the study

The major limitation of this study is the ability to collect sufficient data because of the lack of student compliance. The questionnaire was sent through the list serve of medical students via information technology units at the respective universities. Thousands received the questionnaire, but only 849 responded to it.

## Declarations

### Author contribution statement

Qasim A. El-Dwairi; Ayman G. Mustafa: Conceived and designed the experiments; Performed the experiments; Wrote the paper.

Intisar Mustafeh; Moawiah Khatatbeh; Mohammed I. Malki: Analyzed and interpreted the data; Wrote the paper.

### Funding statement

This study was supported by the Deanship of Research at 10.13039/501100004035JUST (2015/513). Dr. Ayman Mustafa is currently at leave from JUST.

Open access funding provided by the Qatar National Library.

### Data availability statement

Data will be made available on request.

### Declaration of interest’s statement

The authors declare no conflict of interest.

### Additional information

No additional information is available for this paper.
