# Supplementary material for: Medical education across three colleges of medicine: perspectives of medical students
Source: Heliyon. 2022 Nov 5;8(11):e11426. doi: 10.1016/j.heliyon.2022.e11426 (PMC9647486; doi:10.1016/j.heliyon.2022.e11426)
Supplement: Appendix A — . [file mmc1.docx]

**Medical education across three colleges of medicine: Perspectives of medical students**

**Part I: Demographic data**

1. Nationality

- Jordanian
- Other Arabic nationality:…………..
- Other nationality:………………..

1. Age :……………..yrs
2. Gender:

- Female
- Male

1. High school curriculum

- Jordan national curriculum
- Other Arabic national curriculum
- International curriculum

1. University

- Jordan University
- Yarmouk University
- Jordan University of Science and Technology

1. Which year are you at present

- 2^nd^ Year
- 3^rd^ Year
- 4^th^ Year and above

1. What is the nature of your admission

- Regular
- Parallel
- International

1. Why did you choose to study medicine

- Motivation
- Family pressure
- Social Prestige

1. What is your Average

- 60-69
- 70-79
- 80-89
- 90-100

**Part II: General information about students learning behavior**

1. What is your grade in Histology

- Below 50
- 50-59
- 60-69
- 70-79
- 80-89
- 90-100

1. What is your grades in General Anatomy

- Below 50
- 50-59
- 60-69
- 70-79
- 80-89
- 90-100

1. How do you describe your attendance

- Rare
- Irregular
- Regular

1. How do you describe your studies pattern

- Daily
- Weekends
- Before exams

1. What are the sources that you study from for exams

- Lecture notes only
- Lecture notes and textbooks
- Private tutors
- Samples of old exams

**Part III: Students feedback on medical education process at their colleges**

1. Do you think that your Instructors make a positive influence on your academic achievement?

- No
- Some of them
- Most of them

1. Do your Instructors keep their lectures up-to-date in both content and style?

- No
- Some of them
- Most of them

1. Do your Instructors present during their lectures additional information from relevant research articles?

- No
- Some of them
- Most of them

1. Do your Instructors deliver integrated type of lectures

- No
- Some of them
- Most of them

1. Do your Instructors use flipped classroom style

- No
- Some of them
- Most of them

1. Do you think that laboratories sessions enhance your understanding of the relevant topics?

- No
- Some of them
- Most of them

1. Do you think that small group discussion sessions enhance your understanding of the relevant topics?

- No
- Some of them
- Most of them

1. Do you think that the facilitators of small group discussions have a positive influence on your understanding of the relevant topics?

- No
- Some of them
- Most of them

1. How do you rate your satisfaction with the facilities and academic environment in your college?

- Insufficient
- Moderate
- High

1. As a medical trainee what is the number of students in each batch during your clinical rounds?

- Less than 10
- 10 to 20
- More than 20

1. How do you rate your satisfaction with the clinical rounds as a learning venue?

- Insufficient
- Moderate
- High

1. Do your clinical Instructors present during their lectures additional information from relevant research articles and/or clinical trials?

- No
- Some of them
- Most of them

1. Is there any option to participate in active research in your college?

- None
- Occasional
- Available
